# The impact of cation concentration on *Microcystis* (cyanobacteria) scum formation

**DOI:** 10.1038/s41598-019-39619-y

**Published:** 2019-02-28

**Authors:** Bogdan Drugă, Doriana-Mădălina Buda, Edina Szekeres, Ciprian Chiş, Iuliana Chiş, Cosmin Sicora

**Affiliations:** 1 TU Darmstadt, Institute IWAR, Chair of Wastewater Engineering, Franziska-Braun-Straße 7, 64287 Darmstadt, Germany; 2 NIRDBS, Institute of Biological Research, 48 Republicii street, 400015 Cluj-Napoca, Romania; 3 Babeș-Bolyai University, Faculty of Biology and Geology, Department of Molecular Biology and Biotechnology, 5-7 Clinicilor Street, 400006 Cluj-Napoca, Romania; 4 Biological Research Center, 11 Parcului Street, 455200 Jibou, Romania

## Abstract

Cyanobacterial scums at the surface of the lakes are potentially harmful phenomena with increasing occurrence in the last decades, and the causes that lead to their formation are still an unresolved issue. In order to better understand what triggers the scums, we investigated the effect of several Mg^2+^ and Ca^2+^ ion concentrations in promoting them in eight *Microcystis aeruginosa* strains. The possibility to prevent scum formation by using the ion chelator EDTA was also explored. We found that in some strains the cell aggregation takes place under lower ion source concentrations (20 mM MgSO_4_ or CaCl_2_), while in others this phenomenon does not occur even at 60 mM concentration. The scum formation correlated to the amount of extracellular polymeric substances (between 234 and 351 µg/cell). EDTA failed to prevent the scum formation in most strains, and in turn it caused cell lysis followed by the release of cellular content into the culture medium. We emphasize the relevance of these results for cyanobacterial scum formation in the environment and we also suggest that controlling the salinity of the medium (by manipulating the ion concentration) is a potentially efficient method for biomass harvesting in large ponds/tanks.

## Introduction

One of the most important issues that is yet to be fully understood in aquatic ecology is the connection between environmental factors (e.g. temperature, light) and the stability of ecosystems, which is increasingly affected by eutrophication^1^. Among the strongest indicators of freshwater natural environment degradation are the cyanobacterial blooms, which represent excessive accumulation of cyanobacterial cells in the water, generating characteristic scums at water surface under certain conditions^2–4^. In such a scum, the phytoplankton density near the surface can increase by several orders of magnitude within one day, from less than 10^2^–10^3^ cell mL^−1^ up to 10^5^–10^9^ cell mL^−1^ ^2^.

Cyanobacterial scums are harmful for multiple reasons: they block the sunlight for other phytoplankton and plants, they indirectly cause local oxygen depletion in water below, and they can also release toxins (called cyanotoxins) which are harmful for humans and terrestrial or aquatic animals^5,6^. There have been many attempts to understand the process of cyanobacterial scum formation, in order to develop better management solutions for freshwater bodies across the world^7–9^. It was shown that scums are triggered by a combination of several factors: preexistence of a bloom with a high density of cells which also contains buoyant colonies, and a stratified/stable water column^10,11^. Several hypotheses for scum formation were also suggested: the formation of additional gas vesicles^12^, carbon depletion^13^ or self-shading of the cells^14^. Other authors focused their study on the mechanism of scum persistence, and showed that although cyanobacterial gas vesicles are probably not involved in the process, oxygen is needed for cell lifting, and a small increase in water pressure can make the scum to sink (following to gas vesicles disintegration)^15^.

Here, the formation of cyanobacterial scums under different Ca^2+^ and Mg^2+^ cation concentrations was analyzed. These are major ions found in highly variable concentrations in freshwaters, and their presence impacts on phytoplankton metabolism^16^. Nevertheless, there are limited studies aiming at examining the effect of these ions on cyanobacterial scum formation, while cell flocculation mediated by ions present in the environment was previously described both in eukaryotes (yeast) and prokaryotes^17,18^. Collective migration of cyanobacterial cells towards water surface requires the presence of light, extracellular polymeric substances (EPS) and divalent cations (e.g. Mg^2+^ or Ca^2+^) in the medium^19^. Hence, ion chelators could prevent the accumulation of cyanobacterial cells at water surface, with ethylenediaminetetraacetic acid (EDTA) being a good candidate to bind the Mg^2+^ and Ca^2+^ ions in natural and laboratory environments^20,21^. So far, only long-term laboratory adapted *Microcystis aeruginosa* strains were used to study the effect of ions on scum formation^19^, but their physiology may differ from natural strains. It is known that *Microcystis* sp. strains cultivated in the laboratory lose the ability to form colonies after a period of time, as a consequence of producing less EPS^22^. In contrast, natural *M. aeruginosa* displays complex dynamics due to their EPS content that may compete with the upwards migration. For example, EPS production in cyanobacteria is affected by salt stress, nutrient availability or grazing^23–25^, while mutants unable to produce EPS are less tolerant to elevated salt concentrations^26^. Therefore, it would be relevant to study the cyanobacterial scum formation with wild strains isolated from lakes/ponds, which are not affected by decades of laboratory effect.

In this paper, the transition from blooms to scums (the process of scum formation itself) in eight recently isolated *M. aeruginosa* strains was analyzed. The strains were isolated from various freshwaters across Romania, and they were previously described in terms of molecular phylogeny and toxic potential^27^. The main hypothesis was that different strains, although belonging to the same species, would display divergent behaviors when exposed to certain conditions, both for triggering or preventing scum formation. Therefore, the focus was on exploring the effect of divalent cations on this process, but also on the possibility of using ion chelators to prevent scum formation. EPS concentration and chlorophyll fluorescence were analyzed in order to better explain the effect of ions on cell physiology. This study is important for the better understanding of scum dynamics starting from fresh multistrain cyanobacterial communities (multiple strains that could react differently to various ion concentrations). It is environmentally relevant, because cyanobacterial scums often contain toxin-producing strains, with toxins being released in the water after the decay of the scum. Therefore, verifying whether ion concentration selects for certain strains over others in terms of surface migration might improve our understanding of the mechanism of scum formation. It could also make it possible to predict the structure of a potential scum based on the cyanobacterial community in the water. The results might also be relevant from an industrial perspective, as a potential way to stimulate cell aggregation for collecting the biomass from large ponds, which is currently done mostly by expensive centrifugation or filtration of large volumes.

## Results

### Role of cell density in triggering scums

**Figure 1:**
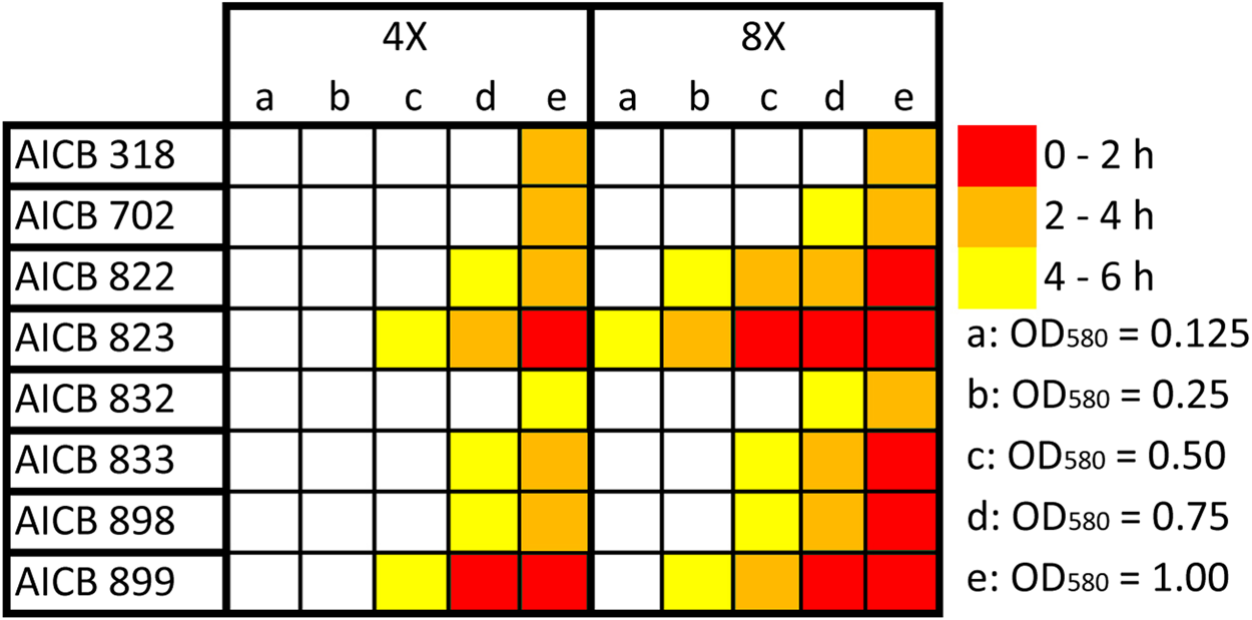
Illustration of scum formation in relation with the initial cell density (OD_580_), in two BG11 concentrations (4x and 8X). Open squares indicate that no cell migration was observed within 6 hours, while solid squares show that scums were generated. Every experiment was done in three replicates.

The minimum growth medium concentration required for scum formation was four times the concentration of standard BG11 (Fig. 1). The lowest optical density at which cells started to migrate upwards was OD_580_ = 0.125, corresponding to a cell density of approx. 1.6 × 10^6^ cell mL^−1^. This was observed in only one strain: AICB 823 (“AICB” stands for the Romanian translation of: “Algae: Institute of Biological Research”), in eight times concentrated BG11 (Fig. 1). In this case scum formation occurred 4 to 6 hours after inoculation. Consistently, more concentrated medium triggered the scum formation faster: at eight times standard BG11concentration, all the tested *M. aeruginosa* strains generated scums, however at different cell densities. Thus, in some strains the migration occurred at lower cell densities: 1.6 × 10^6^ cell mL^−1^ (OD_580_ = 0.125) in AICB 823 and 3.8 × 10^6^ cell mL^−1^ (OD_580_ = 0.25) in AICB 899. Nevertheless, three strains (AICB 318, 702 and 832) produced scums only at high cell concentrations: 1.4 × 10^7^cell mL^−1^ (OD_580_ = 0.75), and 1.8 × 10^7^ cell mL^−1^ (OD_580_ = 1.00) (Fig. 1). The only potentially toxic strains in this experiment (AICB 318 and AICB 702) required very high initial cell density in order to generate scums. The differences in response of the strains persisted also in terms of duration before the upwards migration was observed, and it varied with the strain analyzed and cell density: from 0–2 hours in denser cultures, up to 4–6 hours in the less dense ones.

### Importance of light in scum formation

Under light, the collective migration of the cells towards surface started with the formation of gas bubbles inside some of the cultures 20 minutes after the experiment began. In two of the cultures which were kept in the dark (AICB 823 and AICB 899), cell migration also occurred, but much slower as compared to the light treatment. In these cultures, less gas bubbles were observed as compared to the cultures exposed to light. In the other six strains, no cell migration was observed in the dark.

### Effect of the ions in the scum formation in individual *M. aeruginosa* strains

**Figure 2:**
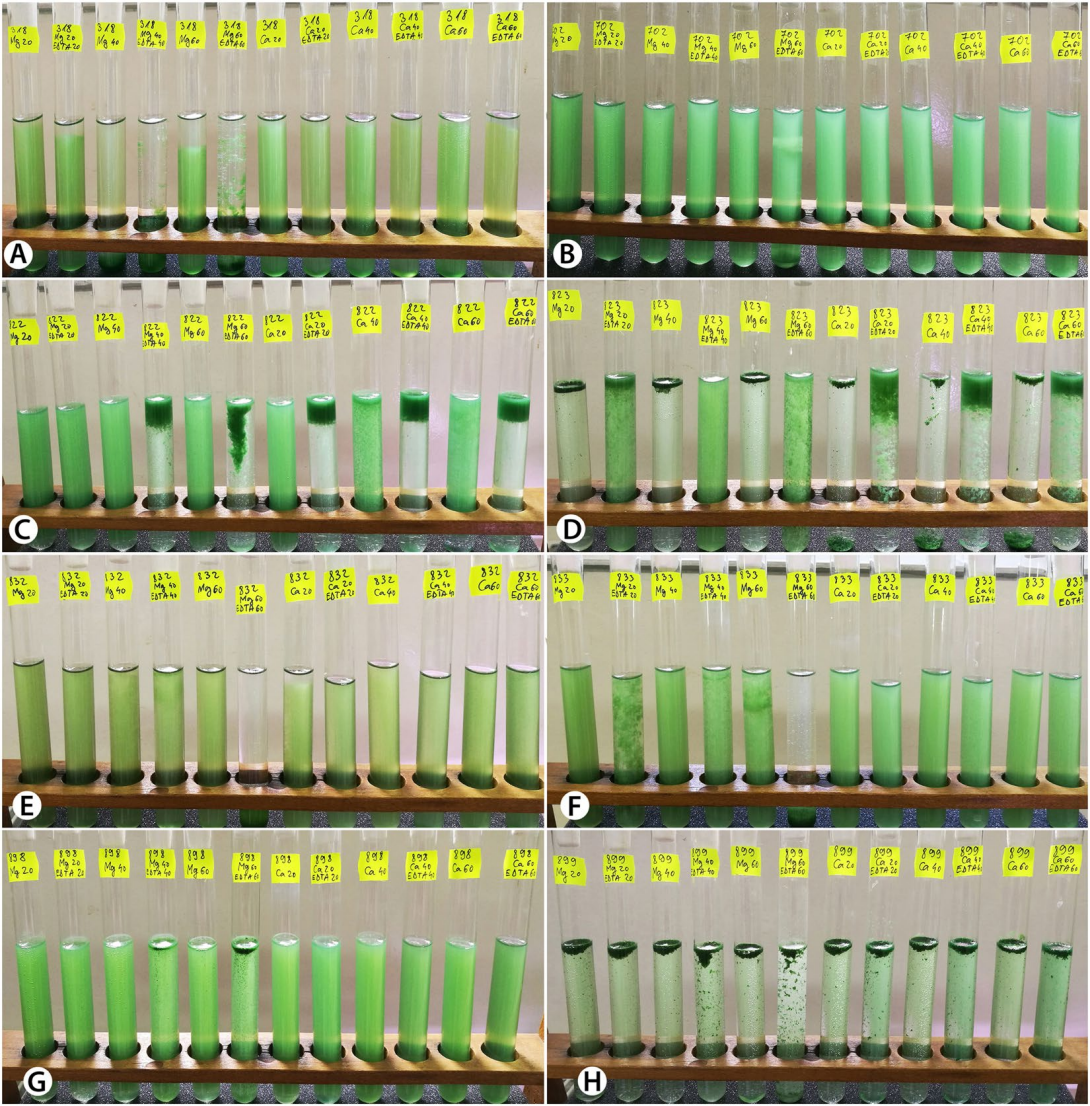
Images with the eight *M. aeruginosa* cultures after 120 minutes of treatment with cations/EDTA. (**A**) AICB 318; (**B**) AICB 702; (**C**) AICB 822; (**D**) AICB 823; (**E**) AICB 832; (**F**) AICB 833; (**G**) AICB 898; (**H**) AICB 899.

In order to better understand the role of the Mg^2+^ and Ca^2+^ cations in the upwards migration of the cells, their influence was tested separately on each *M. aeruginosa* strain. We found that ions have divergent effects on different strains, and that they cause evident cell migration in only two of the eight genotypes: AICB 823 and AICB 899 (Fig. 2D,H). In these two strains, the scum formation was triggered by both ions, and it started after less than 20 minutes of light exposure. A sequential set of images of strain AICB 823 during the 120-minute experiment can be seen in supplementary Fig. S1. Higher concentrations of Mg^2+^ had the opposite effect on strain AICB 318, where cells were slowly sinking after 120 minutes of treatment (Fig. 2A). In the other five strains (including two potentially toxic), addition of ions alone, without EDTA, had no obvious effect on cell migration (Fig. 2B,C,E,F,G).

The effect of Mg^2+^ as compared to control cultures was significant (*p* < 0.05) in three strains: AICB 318, AICB 823 and AICB 899, while the Ca^2+^ ions impacted on four strains: AICB 318, AICB 822, AICB 823 and AICB 899. The effect of two ions was significantly different in three strains: AICB 318, AICB 822 and AICB 833 (Table 1).

**Table 1:** *p* value (Student’s t-test) showing the statistic significance of observed results.

p value	AICB 318	AICB 702	AICB 822	AICB 823	AICB 832	AICB 833	AICB 898	AICB 899
Mg/control	<**0.05**	0,2560	0,2971	<**0.001**	0,0845	0,0629	0,5	<**0.001**
Ca/control	<**0.001**	0,1732	<**0.05**	<**0.001**	0,0630	0,5	0,5	<**0.001**
Mg/Ca	<**0.05**	0,5	<**0.05**	0,5	0,0831	<**0.05**	0,5	0,5
(Mg + EDTA)/(Mg − EDTA)	<**0,05**	0,0845	<**0.05**	<**0.001**	<**0.05**	<**0.05**	<**0.05**	0,1733
(Ca + EDTA)/(Ca − EDTA)	0,1497	0,0845	<**0.001**	<**0.001**	0,0630	0,5	0,5	0,1733
(Mg + EDTA)/(Ca + EDTA)	<**0.05**	0,5	<**0.05**	<**0.001**	<**0.05**	<**0.05**	<**0.05**	0,2234

*Mg*/*control* = strains treated with MgSO_4_ as compared to control cultures; *Ca*/*control* = strains treated with CaCl_2_ as compared to control cultures; *Mg*/*Ca* = strains treated with MgSO_4_ as compared to those treated with CaCl_2_; *(Mg* + *EDTA)*/*(Mg* *−* *EDTA)* = strains treated with MgSO_4_ and EDTA as compared to those treated with MgSO_4_ but without EDTA; *(Ca* + *EDTA)*/*(Ca* *−* *EDTA)* = strains treated with CaCl_2_ and EDTA as compared to those treated with CaCl_2_ but without EDTA; *(Mg* + *EDTA)*/*(Ca* + *EDTA)* = strains treated with MgSO_4_ and EDTA as compared to those treated with CaCl_2_ and EDTA.

**Figure 3:**
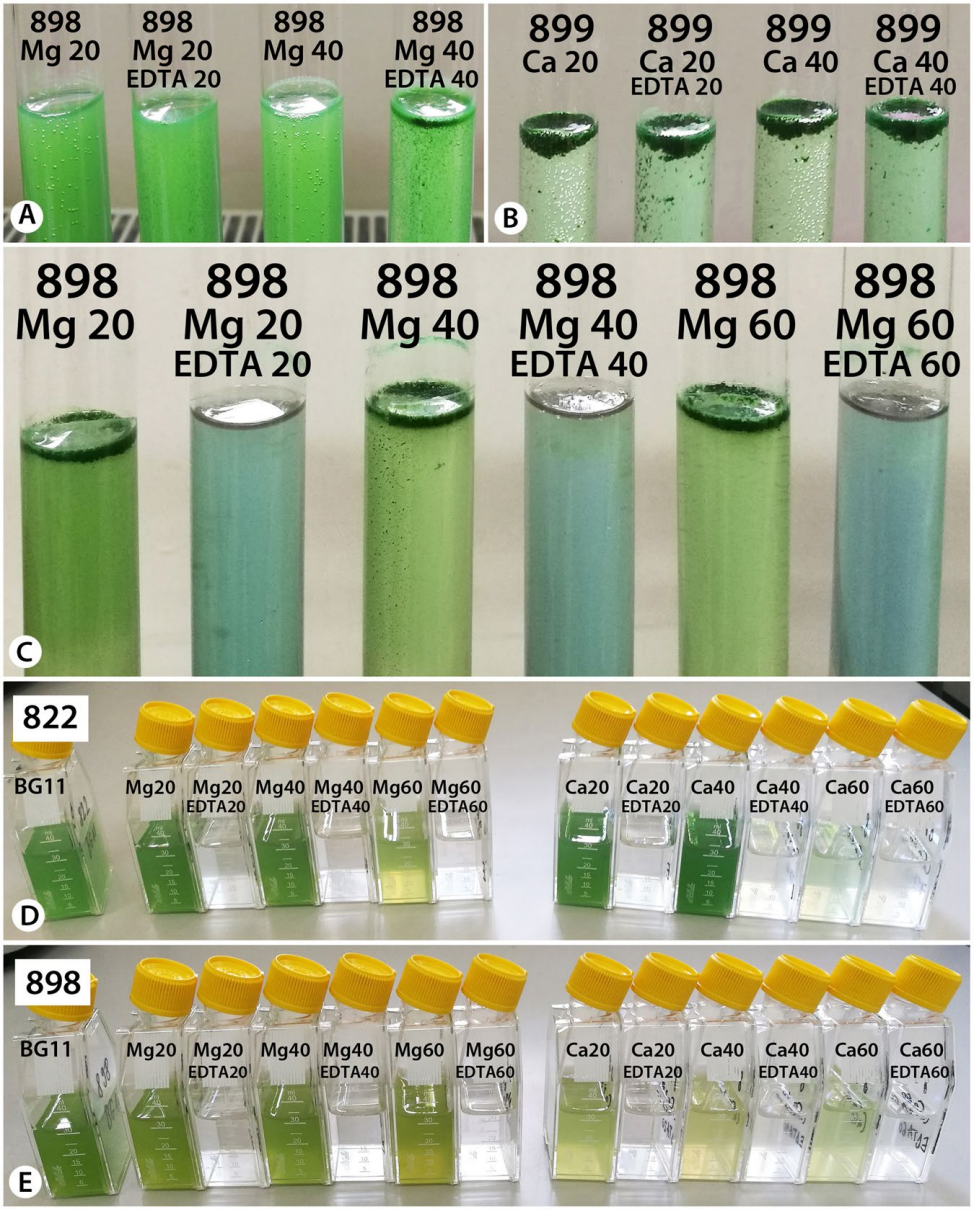
The effect of EDTA on *M. aeruginosa* strains. (**A**,**B**) note the gas bubble presence/absence between cultures with/without EDTA, after 120 minutes of treatment. (**C**) difference in culture color in the presence/absence of EDTA, after 24 hours of treatment. (**D**,**E**) AICB 822 and AICB 898 *M. aeruginosa* cultures 14 days from inoculation with cells previously exposed/not exposed to EDTA.

Previous results have emphasized that the production of oxygen bubbles by cells via photosynthesis is directly connected to scum formation^19^. Similarly, the production of gas bubbles was observed in the strains tested here, in the presence of Mg^2+^ and Ca^2+^ ions, but only in the absence of EDTA (Fig. 3A,B).

### Effect of EDTA on scum formation

The ion chelator EDTA was tested to verify whether it could prevent upwards cell migration by chelating the ions, hence stopping the process of scum formation. No clear pattern was observed, since cell position in the cultures varied between strains. Scum formation was prevented in only one strain: AICB 823, where the cell upwards migration was slower in the presence of EDTA + Mg^2+^ ions as compared to the ones in the presence of ions only (Fig. 2D). The opposite effect was observed in strains AICB 822 and AICB 898, where the presence of EDTA and cations has resulted in cell accumulation in the upper part of the test tubes (Fig. 2C,G). In other strains, in the presence of higher Mg^2+^ concentrations, EDTA has caused the cells to sink to the bottom of the test tubes (Fig. 2A,E,F), while in the remaining strains it had no noticeable effect on cell migration as compared to the test tubes containing only cations (Fig. 2B,H). Overall, adding EDTA had a significant effect on cell upwards or downwards migration in six strains when Mg^2+^ ions were also present, while this effect was only obvious in two strains in the presence of Ca^2+^ ions. It was also observed that when EDTA is present, the effect of Mg^2+^ ions on scum formation is significantly different from Ca^2+^ ions (Table 1).

After the 120-minute experiment, test tubes were kept on the laboratory bench for 24 hours. Following this, another difference between the cultures with/without EDTA was observed: in the EDTA-containing test tubes the color of the growth medium has shifted into pale blue (Fig. 3C), suggesting cell lysis and cyanobacteria blue pigments release in the medium (phycocyanin and allophycocyanin). A subsequent control experiment where only EDTA was added to fresh cultures had the same result, after 24 hours the cells being located at the bottom of the tube, with the medium turning blue. This damaging effect on cells was also confirmed by the fact that no gas bubbles (oxygen produced during photosynthesis) were observed in the tubes containing EDTA (Fig. 3A,B). Moreover, the cultures inoculated with cells after 24 hours of exposures to cations ± EDTA were viable after 14 days only when the inoculum did not contain EDTA (Fig. 3D,E).

### Effect of EPS concentration

**Figure 4:**
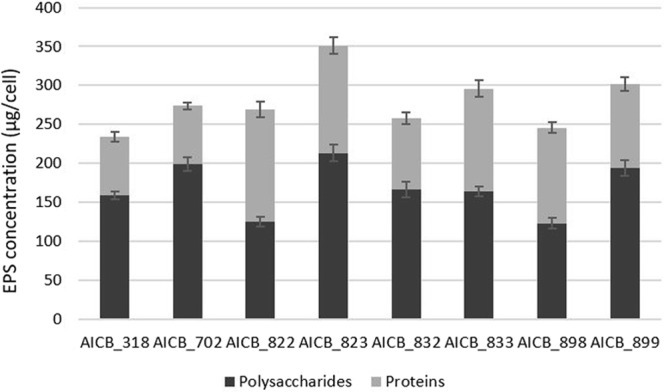
EPS composition and concentration in the eight *M. aeruginosa* strains.

The EPS concentration varied between 234 and 351 µg/cell among the eight strains, with an average of 279 µg/cell (Fig. 4). The polysaccharide fraction was in average 60% of the total EPS, while the protein fraction was of approx. 40%.

**Figure 5:**
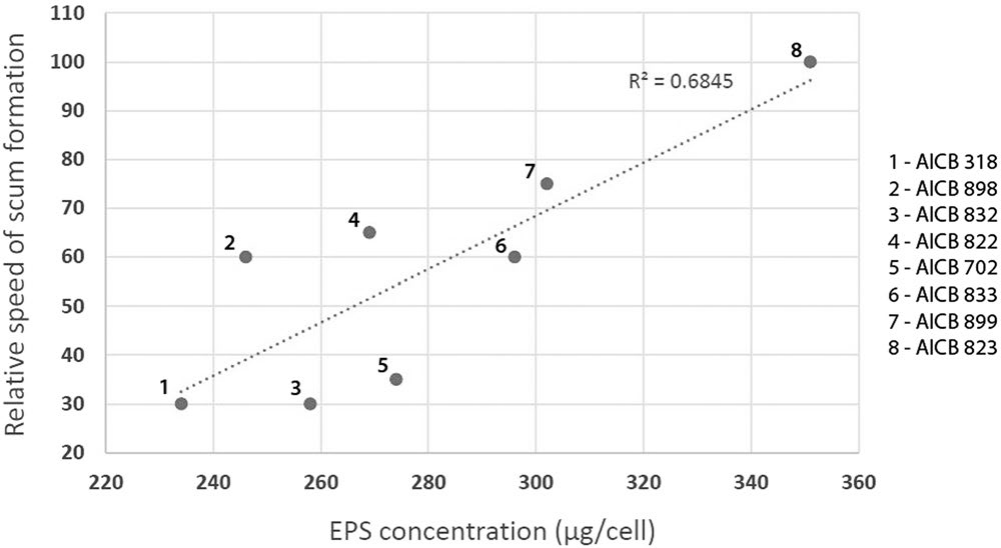
Relative speed of scum formation plotted against EPS concentration in eight *M. aeruginosa* strains. The speed of cell upwards migration was calculated relative to strain with the fastest scum formation (AICB 823), which was considered 100%.

Strain AICB 822 was the only one with a higher fraction of proteins (53%) than polysaccharides (47%). The differences between the strains are statistically significant (p ≤ 0.05), with one exception: strains AICB 318/AICB 832, with a p-value of 0.076. Furthermore, plotting EPS concentration against the speed of scum formation showed a positive correlation between the two parameters (Fig. 5).

### Chlorophyll fluorescence

Changes in the number of actual active and potential active photosynthetic centers (in the absence and presence of DCMU) were measured. By adding DCMU (3-(3′,4′-dichlorphenyl)-1,1-dimethylurea) to the cells, the electron transfer in PSII complex is blocked, thus bringing all centers into the same physiological state and allowing the measurement of the total potential active centers. Both in the presence and absence of DCMU, the results clearly showed an abrupt reduction in the number of active PSII centers in the cultures where EDTA was added. In these samples, the relative fluorescence has decreased up to 90% compared to the cells exposed to cations only (Supplementary Figs S2 and S3). The fluorescence analyses have revealed different effects of Mg^2+^ and Ca^2+^ ions on the functioning of the photosynthetic apparatus. For example, the higher concentrations of Mg^2+^ (40, 60 mM) have greatly reduced the number of both active and potentially active photosynthetic centers in strain AICB 822 as compared to the lower concentration (20 mM) (Supplementary Fig. S2A,B), while the effect of Ca^2+^ was more constant between different concentrations (Supplementary Fig. S2C,D). On the contrary, in strain AICB 832 the effect of Mg^2+^ was similar with Ca^2+^, with the number of photosynthetic centers slowly decreasing with time (Supplementary Fig. S3A–D). In the other six strains, the impact of the two ions ranged between the results measured in the two above-mentioned strains.

## Discussion

*Microcystis* sp. scums are a worldwide serious threat for water quality, ecosystems, fisheries, and human health because this widespread species is also potentially toxic. Understanding cyanobacterial surface scum formation is of a considerable importance as these compact aggregates are responsible for the serious effects associated with cyanobacterial blooms: toxin release in the water, decrease of light availability for aquatic plants below the surface, unpleasant aspect and odor etc.^4^. The doubling time of most cyanobacteria (usually several days) cannot explain the development of scums (usually less than 1 day). The main objective of this research was to investigate whether the presence of different Mg^2+^ and Ca^2+^ ion concentrations in the growth medium would impact on the process of scum formation in multiple cyanobacterial strains. Particularly, the transition from cyanobacterial blooms to scums in the presence of ions was explored, focusing on eight recently-isolated strains belonging to the potentially toxic species *M. aeruginosa*.

The conditions used in this study are environmentally relevant, being situated in a range found in natural aquatic ecosystems for light, nutrients and salinity^28,29^. Even though Ca^2+^ and Mg^2+^ concentration in eutrophic/hypertrophic lakes are, in average, lower than the ones used here, there are plenty of data reporting very high concentrations for these ions in the environment^30,31^. Some of the strains studied here generated scums in less than 20 minutes following ion source addition, a process likely to happen in nature at lower salt concentrations, even if that might take longer time.

Initial cell density greatly affects the upward cell migration. In denser cultures cells lifted towards the surface much faster than in the less dense ones (Fig. 1). This can be explained by enhanced photosynthetic activity in dense cultures leading to O_2_ saturation^19^ and nucleation into bubbles (Fig. 3A,B). Bubbles inside colonies/cell aggregates are assumed to have grown starting from microbubbles arising from heterogeneous nucleation. When this process take place, O_2_ bubbles, trapped inside EPS, lift most of the biomass to the surface, forming a dense layer, very much alike to natural blooms^15^. As a result, O_2_ can super-saturate (210%) inside surface scum^32^.

Overall, the strains used here started to migrate at lower cell concentrations as compared to other experiments. This is most likely due to the higher amount of EPS produced by the recently isolated strains as compared to strains grown in the laboratory for decades. The strains used in this study (up to seven years old) produce in average 279 µg EPS per cell (Fig. 4), which is almost double than for *M. aeruginosa* PCC 7005 (isolated in 1946) or PCC 7806 (isolated in 1972)^19^, and therefore promoted the scums faster. Many functional roles of cyanobacterial EPS (colony formation, protection, diffusion of molecules both into and out of the cell) are associated to their physico-chemical properties and explain their function in the physiology of the organisms which include scums or biofilm formation^23–25,33^.

Interestingly, the two toxic strains in this study (AICB 318 and AICB 702) generated scums only at very high cell concentrations, four to six hours after the experiments began (Fig. 1). This suggests that toxic strains could be less favored by ion presence to generate scums. In these two strains, the increased Ca^2+^ and Mg^2+^ concentrations also did not trigger the cell migration, as in some of the non-toxic strains (Fig. 2). This is in agreement with a previous study on a non-toxic and a toxic *M. aeruginosa* strains, where the latter only formed scums at much higher ion concentrations as compared to the non-toxic one^19^. Such findings show that there might be a trade-off between toxin production and the scum formation potential of the strains. Recent studies have suggested that factors like pH or temperature have distinct effects on toxic/non-toxic *M. aeruginosa* genotypes^34,35^. Nevertheless, our results were statistically significant only for one of the two potentially toxic strains (AICB 318) (Table 1), and therefore they should be interpreted with caution. Even though a connection between toxic potential and scum formation is plausible, it requires further investigations, with more toxic/non-toxic strains.

Overall, it was concluded that different Ca^2+^ and Mg^2+^ concentrations might affect the scum formation of various cyanobacterial strains, by selecting certain types from a pool of multiple genotypes. The Mg^2+^ and Ca^2+^ ions impacted in different ways on the position of the cells in the liquid column, especially when EDTA was also added (Table 1). It was difficult to identify a pattern, since in some strains the cells quickly accumulated at the surface, in others they started to sink, while in some strains there was no obvious effect on scum formation (Fig. 2). Moreover, in two strains whose scum formation was significantly impacted by ions as compared to controls (AICB 823 and AICB 988), no substantial difference between the effects of the two ions was observed (Table 1). The opposite result (no major effect of individual ions on scum formation relative to control cultures, but significant differences between the effect of the two ions) was observed in strain AICB 833 (Table 1). The two strains that generated scums in the presence of ions (AICB 823 and AICB 899) showed also the highest EPS production, which could explain the results (Fig. 4). This is also confirmed by the correlation that we found between the speed of scum formation and the EPS produced by each strain (Fig. 5). These findings are in line with previous studies emphasizing the importance of EPS for the collective cell migration to the water surface, being known that even the strains that are unable of naturally producing EPS will produce scums when these substances are added to the medium^19^. Interestingly, we have also noticed differences between the effects of the two ions in the cultures, especially after EDTA addition. Thus, in strain AICB 823, in the absence of EDTA, adding Ca^2+^ resulted in half of the biomass to accumulate at the surface, and the other half to sink at the bottom of the tubes, which did not happen in the experiments with Mg^2+^ (Fig. 2D). This suggests that, even if cell flocculation threshold usually varies with ion charge^18^, different ions with the same charge have also divergent effects on scum formation (Table 1). This conclusion confirms other results that found that, for example, *Salmonella* cell motility is more affected by Ca^2+^ than by Mg^2+^, and suggests that various cation concentrations impact the biology of a broad range of unicellular organisms^17^. Moreover, these ions affect differently the overall physiology of the cells, by impairing the electron flow across PSII, thus reducing the cell photosynthetic activity, especially at higher ion concentrations (Supplementary Fig. S2).

As other studies have shown, gas vesicles are probably not responsible for scums. Even though all eight strains possess these structures, and all of the experiments started with the same cell concentrations, the results (in terms of scum formation) were different. Using their gas vesicles, *M. aeruginosa* cells can migrate in the water column with ~1 mm/h^36^, while the upwards cell migration observed in our study was well above 1 cm/h (Supplementary Fig. S1). Moreover, Aparicio Medrano *et al*.^15^ have found that a minor increase in water pressure (not enough to disintegrate the gas vesicles, but enough to disrupt O_2_ bubbles trapped within the EPS layer) can change the buoyancy status of cyanobacterial scums, meaning that gas vesicles are less relevant in this process.

Based on this investigation, using ion chelators is unlikely to be an effective way of preventing natural cyanobacterial scum formation. EDTA, which is the most common organic ligand used in microalgae cultivation in both freshwater and seawater media^37^, has prevented scums in only one strain in which ions have previously triggered this phenomenon (AICB 823) while it had no effect against scums in AICB 899 (Fig. 2D,H). Overall, EDTA significantly impacted on cell migration in six out of eight strains when it was present in the medium together with the Mg^2+^ ions (Table 1). Moreover, there was a significant difference between the effect of EDTA + Mg^2+^ as compared to EDTA + Ca^2+^ on cell position in the medium, proving that not only the ion charge is important, but also the ion type. The fact that EDTA could not prevent scum formation in all the other strains may indicate that a higher EDTA concentration might be necessary in the medium. However, since after 24 h of exposure the cells were lysed in the presence of EDTA, and the pigments were released in the medium (Fig. 3C), increasing the EDTA concentration is not advised. Therefore, in a scenario where ion chelators would be released in natural environments to stop scum formation, cells could still gather at water surface, but they would also simultaneously release their contents in the water due to lysis. Considering that cyanobacterial blooms consist in mixed populations, with some strains being able to produce toxins, such an approach could be extremely detrimental for the whole ecosystem. Cell lysis due to EDTA also explains the lack of gas bubbles in the test tubes after 120 minutes of experiment (Fig. 3A,B). It also suggests that these bubbles are in fact O_2_ produced by the cells through photosynthesis (much fewer bubbles were produced in dark, in only two strains), which is required for scum formation, as previously shown. The fact that the fluorescence amplitude was almost down to zero in the cultures treated with EDTA emphasizes the damaging effect that this ion chelator has on cell physiology (Supplementary Figs S2 and S3).

Such laboratory scale experiments have a number of advantages: they allow a rigorous test of clear hypotheses and provide both statistical correctness and accuracy of experimental design. Therefore, they were necessary in this experimental design in order to fully control the desired environmental parameters. On the other hand, the results achieved in the controlled laboratory conditions are not always reproducible in nature, even when working with field-isolated strains. The effect of parameters like wind, precipitations or water stratification are important for scum formation. For example, a wind speed of more than 3 m s^−1^ will produce water turbulences which could interfere with cyanobacterial scum formation^38^. Since now the necessary information is known, the following step to these tests would be to upscale the experiments to lake enclosures (mesocosms), in order to compare the lab results with the natural scenario^39^. Moreover, scum formation at different light intensities (as observed in nature) will be addressed in a following study.

These results have not only an environmental relevance in the context of cyanobacterial scums, but they could also be important from an industrial perspective. Cyanobacteria are known for their potential in biofuel, pigments or even food supplement industries, where they need to be separated from the growth medium. Filtration or centrifugation of large volumes are two examples of such costly options for harvesting the cells. Stimulating the global cell aggregation/migration by carefully selecting certain strains, and by manipulating the salinity/EPS content of the medium (not including EDTA addition, which in this study caused cell lysis) could be a less expensive alternative for collecting the biomass from large tanks/ponds.

## Conclusion

The results of this study indicate that cyanobacterial scum formation in some *M. aeruginosa* strains is substantially influenced by the concentration of divalent cations (Ca^2+^ and Mg^2+^). Nevertheless, the response to the presence of ions in the medium strongly varied among strains, with scums being triggered or prevented under different ion concentrations. The potentially toxic strains were among the last ones to generate scums, suggesting that there could be a trade-off between toxic production and scum formation. Furthermore, the ion chelator EDTA did not always prevent the upwards cell migration. Scum formation was highly correlated to the amount of EPS produced by cells. The results achieved here are important for better understanding the process of scum formation starting from mixed cyanobacterial populations, but also from an industrial perspective, as a potential alternative to stimulate cell aggregation for collecting the valuable biomass.

## Methods

### Biological material and growth conditions

The 8 cyanobacterial strains used in this study (AICB 318; AICB 702; AICB 822; AICB 823; AICB 832; AICB 833; AICB 898 and AICB 899) were isolated over the last 7 years from freshwaters in Romania, and were provided as non-axenic cultures by the Collection of Cyanobacteria and Algae (AICB) of the Institute of Biological Research in Cluj-Napoca, Romania^40^. Their phylogenetic identity was previously assessed based on the 16S rDNA gene sequence, which confirmed that they belong to the *M. aeruginosa* species. In the same study, the toxic potential of the strains was analyzed using the matrix-assisted laser desorption/ionization time-of-flight technique (MALDI TOF-MS), which showed that two of the strains (AICB 318 and AICB 702) produced toxins^27^. The organisms were grown as batch cultures in BG11 medium, pH 7.5^41^, at 22–25 °C and a constant light intensity of 20 μmol m^−2^ s^−1^. Constant light was preferred over a dark/light cycle because the scum formation in nature takes place over a few hours, during day time.

### Biomass measurements

Optical density of the samples was measured at 580 nm^42^ using a Shimadzu UV-1700 spectrophotometer (Kyoto, Japan) with a cuvette having a 1 cm light path. Cell counting was carried out with a Thoma cell-counting chamber using a Nikon TE-2000 U inverted microscope (Tokyo, Japan). After analyzing all strains based on serial dilutions, an average concentration of 1.8 × 10^7^ cell mL^−1^ at an optical density OD_580_ = 1 was calculated.

### Experimental setup

The experiments were performed separately for each strain in BG11 medium, at 25 °C and under constant fluorescent light of 20 μmol photons m^−2^ s^−1^. In order to check the minimum density where cells start to migrate upwards, aliquots from each strain in exponential growth phase (OD_580_ ∼ 1.5) were diluted to reach a range of optical densities of OD_580_ = 1.0; 0.75; 0.5; 0.25 and 0.125. Then, they were re-suspended in concentrated BG11 (up to 8 times the concentration of standard medium) and they were observed for six hours. These values were chosen bearing in mind that the cells of the laboratory strain *M. aeruginosa* PCC 7005 are unable to migrate towards the surface at OD580 < 0.1, in 8 times concentrated BG11^19^.

Based on the results of the above-mentioned test, it was decided for all subsequent experiments to run with aliquots from each strain diluted to optical densities of OD_580_ = 0.9 (~1.6 × 10^7^cell mL^−1^). The goal was to provide each strain with the minimum conditions for scum formation, and this was the lowest cell density at which cell migration was observed (in strain AICB 318). We attempted to trigger the scum formation by separately adding the cation sources: MgSO_4_ (for Mg^2+^) and CaCl_2_ (for Ca^2+^) in concentrations of 20, 40 and 60 mM to the standard BG11 medium. All experiments were performed in three replicates, in test tubes containing 20 mL of culture. The scum formation (time of flocculation) was estimated by photographing each culture with a Nikon D200 camera 20, 40, 60 and 120 minutes after salt addition. Part of the test were done in complete darkness, to investigate the role of light in scum production. The presence of gas vesicles was not manipulated in these experiments, since it was previously shown that scum formation is independent of these structures^15,19^.

In order to verify the possibility to prevent the upwards cell migration, the ion chelator EDTA was added to combine and capture the cations that are crucial by their interaction with extracellular polymeric substances (EPS) in causing the scum formation. It is known that EDTA has an increased buffering effect for Mg^2+^ and Ca^2+^ ions^20,21^, and that an EDTA:ions ratio of over 0.5 is required to bind about half of the ions in the medium^43^. Considering the goal of binding as many ions as possible in order to limit/prevent scum formation, an EDTA:ions ratio of 1 was used in these experiments (20, 40 and 60 mM EDTA). An “EDTA only” control experiment was also carried out, where EDTA was added to the cultures but Mg^2+^ and Ca^2+^ concentrations were not manipulated. The Student’s t-test was used to assess whether the differences observed between strains over time (as compared to control, untreated cultures) are significant (p < 0.05, where p evaluates how well the sample data express variances between measurements).

The effect of increased ion concentrations on cell viability was checked by inoculating 40 mL of BG11 growth medium with 1 mL of cell suspension after 24 hours of ion treatment. These cultures were maintained for 14 days under standard conditions (25 °C and constant fluorescent light of 20 μmol m^−2^ s^−1^), after which they were imaged using a digital camera.

### EPS concentration

The relevance of EPS concentration for the scum formation in each *M. aeruginosa* strain was analyzed as well. EPS were extracted as described elsewhere^24^, using several steps of culture sonication, centrifugation, filtration and ethanol purification. The protein and polysaccharide contents of the EPS were measured using the Sigma TP0300 Total Protein Kit (Sigma-Aldrich, Missouri, USA), following the manufacturer’s instructions, and a phenol-sulfuric assay using glucose as control. All assays were done in three replicates, and Student’s t-test was used to calculate the significance of the variations in protein/polysaccharides content between strains. Next, EPS concentration for each strain was plotted against the relative speed of scum formation, in order to verify whether EPS are relevant to this process.

### Chlorophyll fluorescence

This parameter was measured in order to track any physiological effect of ions/EDTA on the photosynthetic apparatus. Flash-induced intensification and decline of fluorescence was measured using a double-modulation fluorometer (PSI Instruments, Brno, CZ). The measuring flash (2.5 µs) and actinic flash (20 µs) were produced by red LEDs. All the measurements were taken logarithmically in the interval of 150 µs to 100 s, in the presence/absence of the Photosystem II (PSII) inhibitor 3-(3′,4′-dichlorphenyl)-1,1-dimethylurea (DCMU) at a final concentration of 10 µM, in order to block the transfer of electrons throughout photosystems^44^. Analysis of the fluorescence decrease was based on the model of the two-electron gate as described elsewhere^45^. All experiments were done in three replicates, and graphic representation of chlorophyll fluorescence was generated using the data analysis software Origin (OriginLab, Northampton, MA).

## Supplementary information

Supplementary Information

## Data Availability

All data generated or analyzed during this study are included in this published article (and its Supplementary Material).
