# Supplementary material for: The impact of cation concentration on Microcystis (cyanobacteria) scum formation
Source: Sci Rep. 2019 Feb 28;9:3017. doi: 10.1038/s41598-019-39619-y (PMC6395708; doi:10.1038/s41598-019-39619-y)
Supplement: Supplementary file 1 — Supplementary Information [file 41598_2019_39619_MOESM1_ESM.docx]

**The impact of cation concentration on cyanobacterial scum formation**

Bogdan Drugă^1,2^, Doriana-Mădălina Buda^3^, Edina Szekeres^2^, Ciprian Chiş^4^, Iuliana Chiş^4^, Cosmin Sicora^4^

^1^ TU Darmstadt, Institute IWAR, Chair of Wastewater Engineering, Franziska-Braun-Straße 7, 64287 Darmstadt, Germany

^2^ NIRDBS, Institute of Biological Research, 48 Republicii street, 400015 Cluj-Napoca, Romania

^3^ Babeș-Bolyai University, Faculty of Biology and Geology, Department of Molecular Biology and Biotechnology, 5-7 Clinicilor Street, 400006 Cluj-Napoca, Romania

^4^ Biological Research Center, Strada Parcului 11, 455200 Jibou, Romania

**^*^Corresponding author**: Bogdan Drugă, Technical University Darmstadt, Institute IWAR, Chair of Wastewater Engineering, Darmstadt, Germany

Tel.: +49(0) 6151 1620 312; e-mail: [b.druga@iwar.tu-darmstadt.de](mailto:b.druga@iwar.tu-darmstadt.de)

Supplementary Material


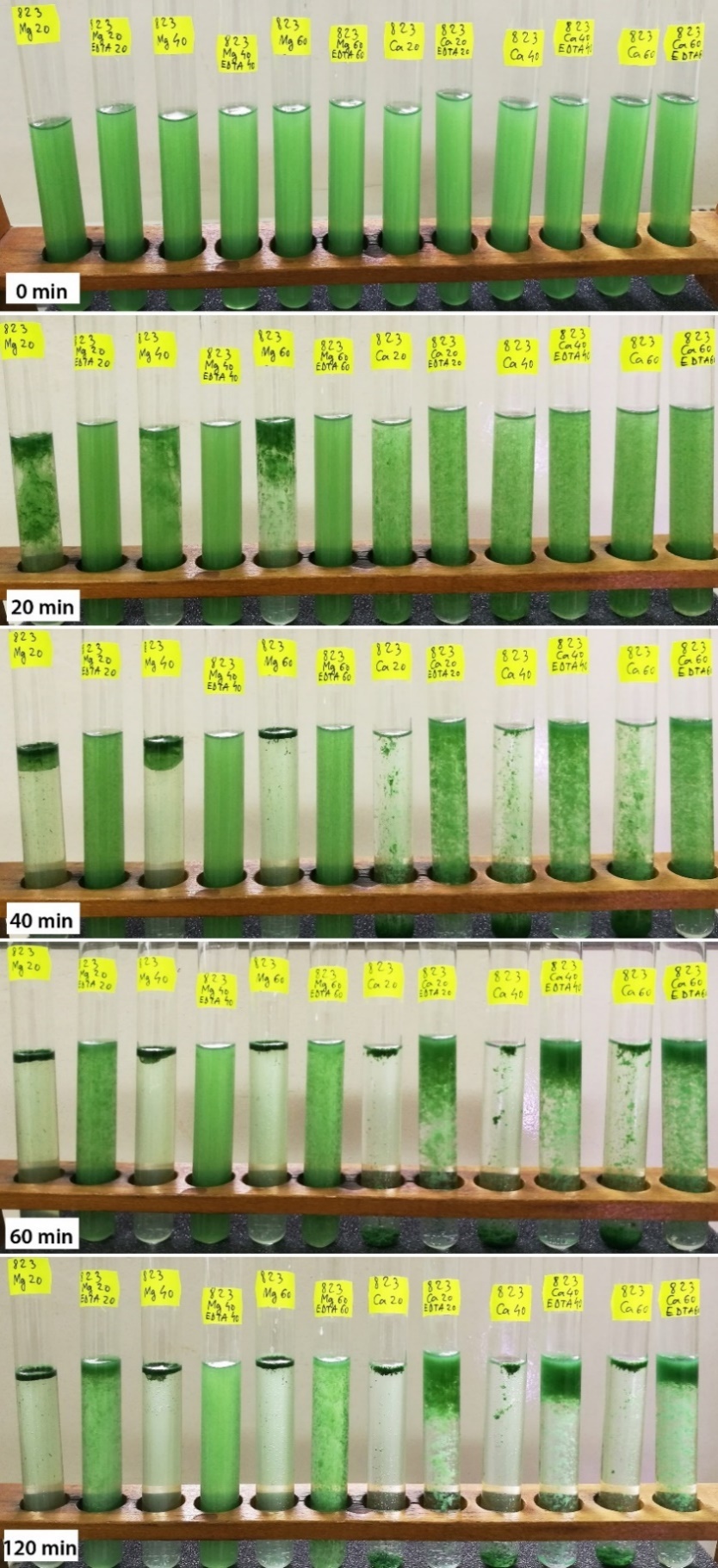


**Suppl. Fig. S1** Sequential set of images of strain AICB 823 during the 120-minute experiment. Note that both Mg^2+^ and Ca^2+^ ions trigger the cell upwards migration, but Ca^2+^ causes the sinking of part of the cells. EDTA prevents this phenomenon, but more in test tubes containing Mg^2+^


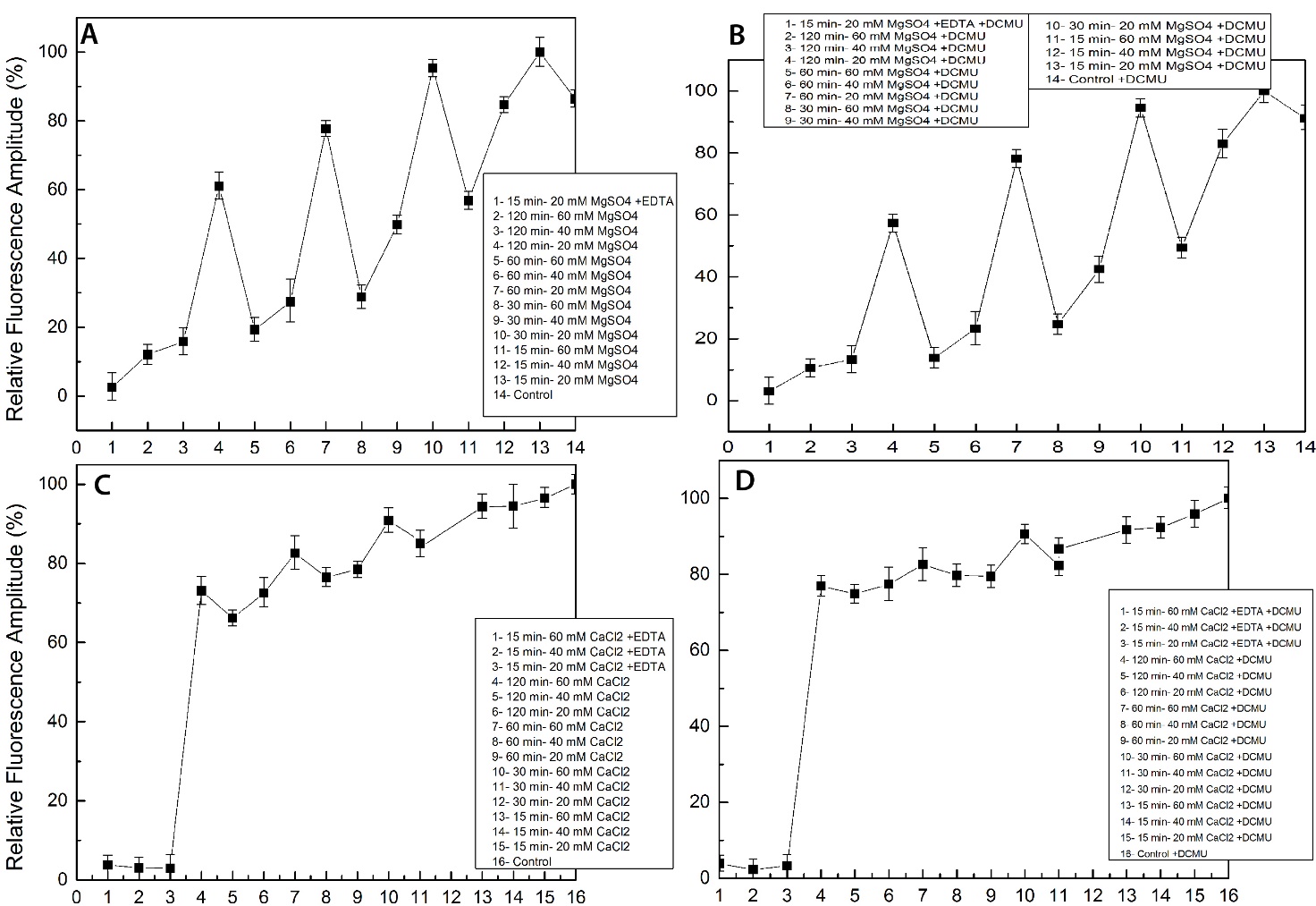


**Suppl. Fig. S2** The effect of different ion concentrations on chlorophyll fluorescence in strain AICB 822. a: Mg^2+^ without DCMU; b: Mg^2+^ with DCMU; c: Ca^2+^ without DCMU; d: Ca^2+^ with DCMU. Data points represent independent measurements of strains undergoing different treatments, and they are shown as being connected only to better represent the differences between experiments


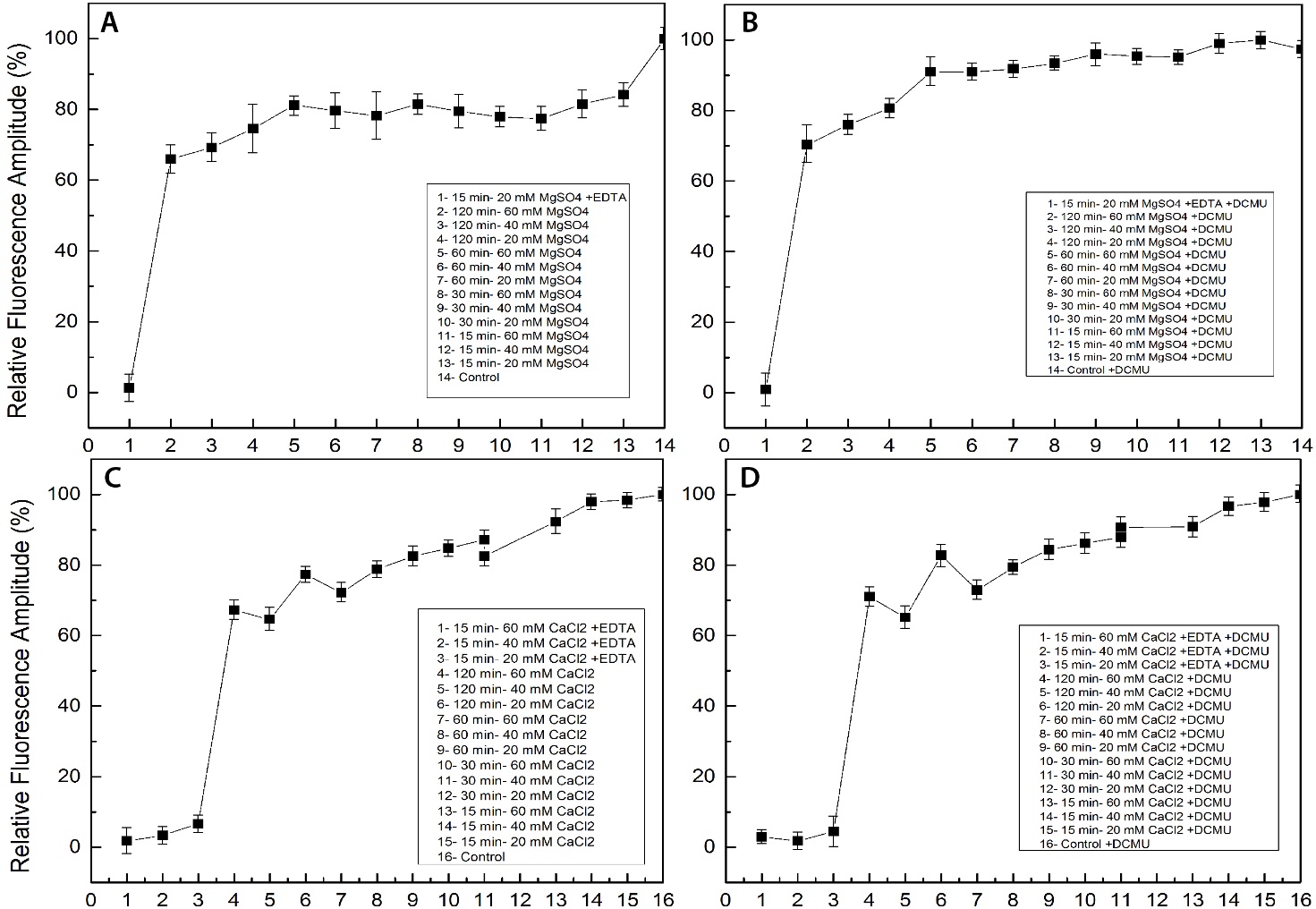


**Suppl. Fig. S3** The effect of different ion concentrations on chlorophyll fluorescence in strain AICB 832. a: Mg^2+^ without DCMU; b: Mg^2+^ with DCMU; c: Ca^2+^ without DCMU; d: Ca^2+^ with DCMU. Data points represent independent measurements of strains undergoing different treatments, and they are shown as being connected only to better represent the differences between experiments
